# Supplementary material for: Carbonyl sulfide (COS) emissions in two agroecosystems in central France
Source: PLoS One. 2022 Dec 6;17(12):e0278584. doi: 10.1371/journal.pone.0278584 (PMC9725148; doi:10.1371/journal.pone.0278584)

ICOS Atmosphere Thematic Centre

CO2 - Vertical gradient

P0034.4.1 /
update 2022-08-23 16:11

2019-01-01 - 2019-12-31

TRN - France

Night (0-4) Dawn (5-11) Day (12-16) Dusk (17-23)

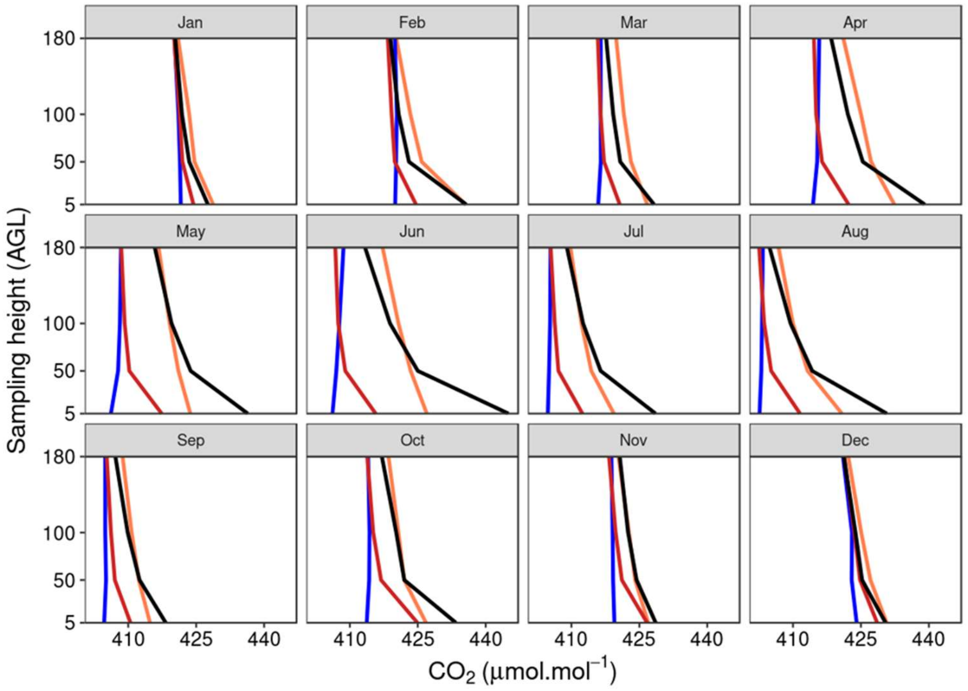

ICOS Atmosphere Thematic Centre

CO2 - Vertical gradient

P0034.4.1 /
update 2022-08-23 16:06

2020-01-01 - 2020-12-31

TRN - France

Night (0-4) Dawn (5-11) Day (12-16) Dusk (17-23)

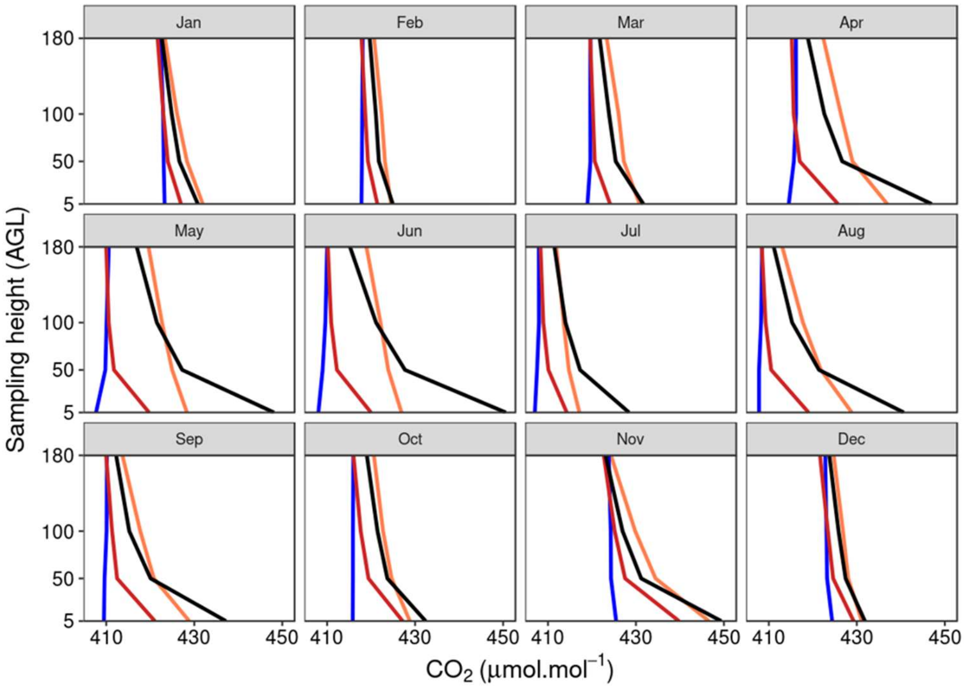

Supplement: S1 Fig — These are standard ICOS products generated by the ICOS database. (PDF) [file pone.0278584.s001.pdf]
