# Supplementary figures and images for: Carbonyl sulfide (COS) emissions in two agroecosystems in central France
Source: PLoS One. 2022 Dec 6;17(12):e0278584. doi: 10.1371/journal.pone.0278584 (PMC9725148; doi:10.1371/journal.pone.0278584)

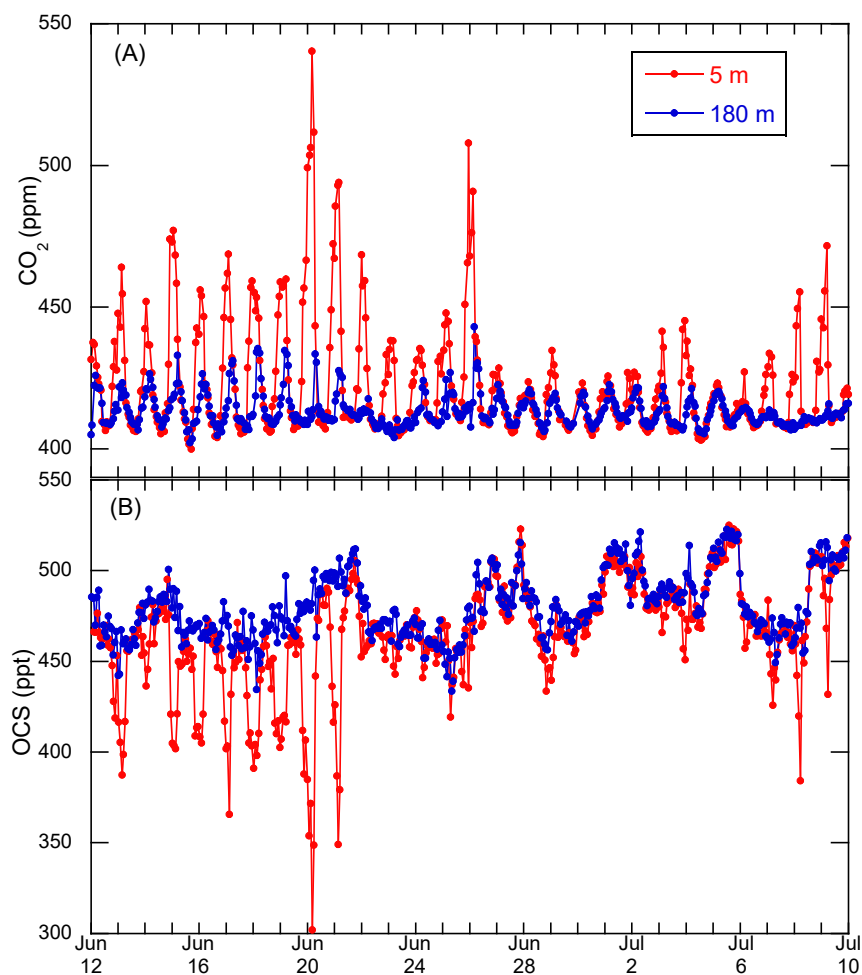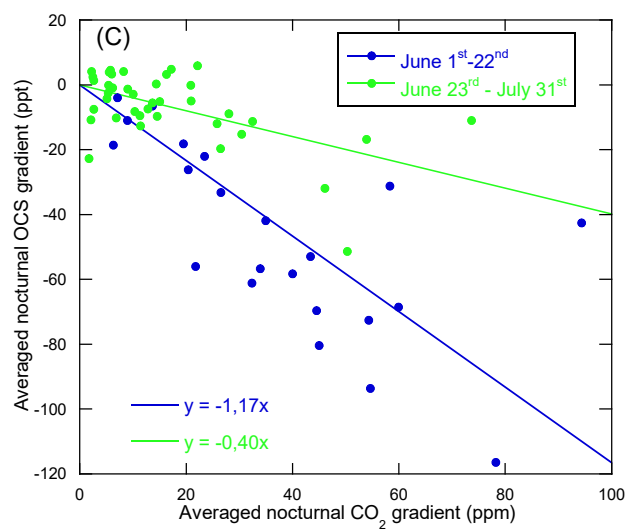

Supplement: S2 Fig — (A) CO2 and (B) COS (OCS) mixing ratios measured at 5 and 180 m roughly on an hourly basis at the TRN site in June-July 2020. (C) Correlation of COS (OCS) and CO2 averaged nocturnal vertical gradients measured on a daily basis. Those linear regressions forced through zero are not calculated strictly on a monthly basis but after data selection because the transition from one regime (slope = -1.17 ppt/ppm) to another (slope = -0.4 ppt/ppm) took place the night of June 21st to 22nd as shown in panels A and B. (PDF) [file pone.0278584.s002.pdf]

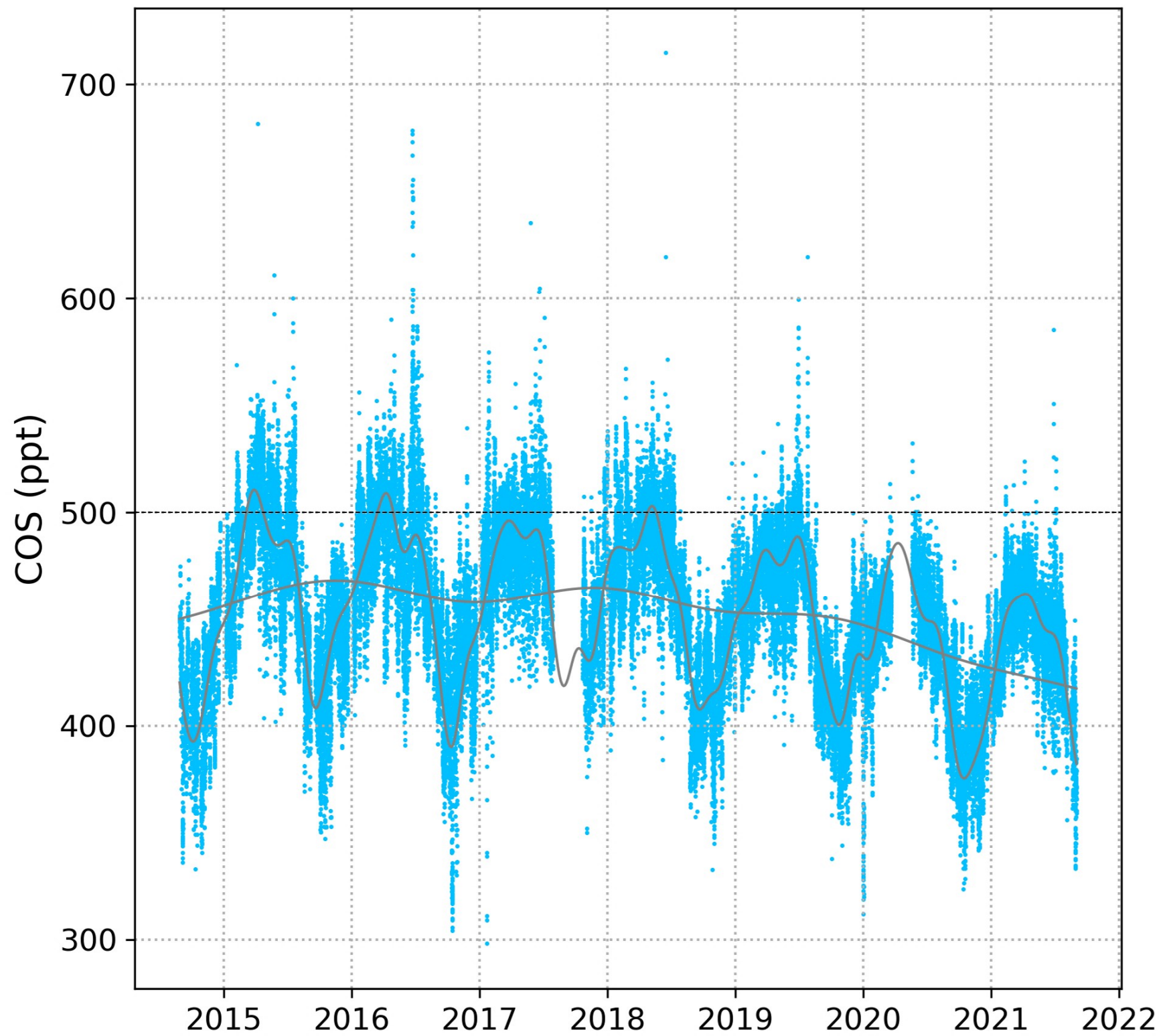

Supplement: S3 Fig — The data gaps in summer/early autumn of 2017 and spring 2020 being a failure of the Entech preconcentrator and the consequence of the French lockdown, respectively. The full COS records are now available from https://doi.org/10.14768/6800b065-dcec-4006-ada5-b5f62a4bb832. (PDF) [file pone.0278584.s003.pdf]

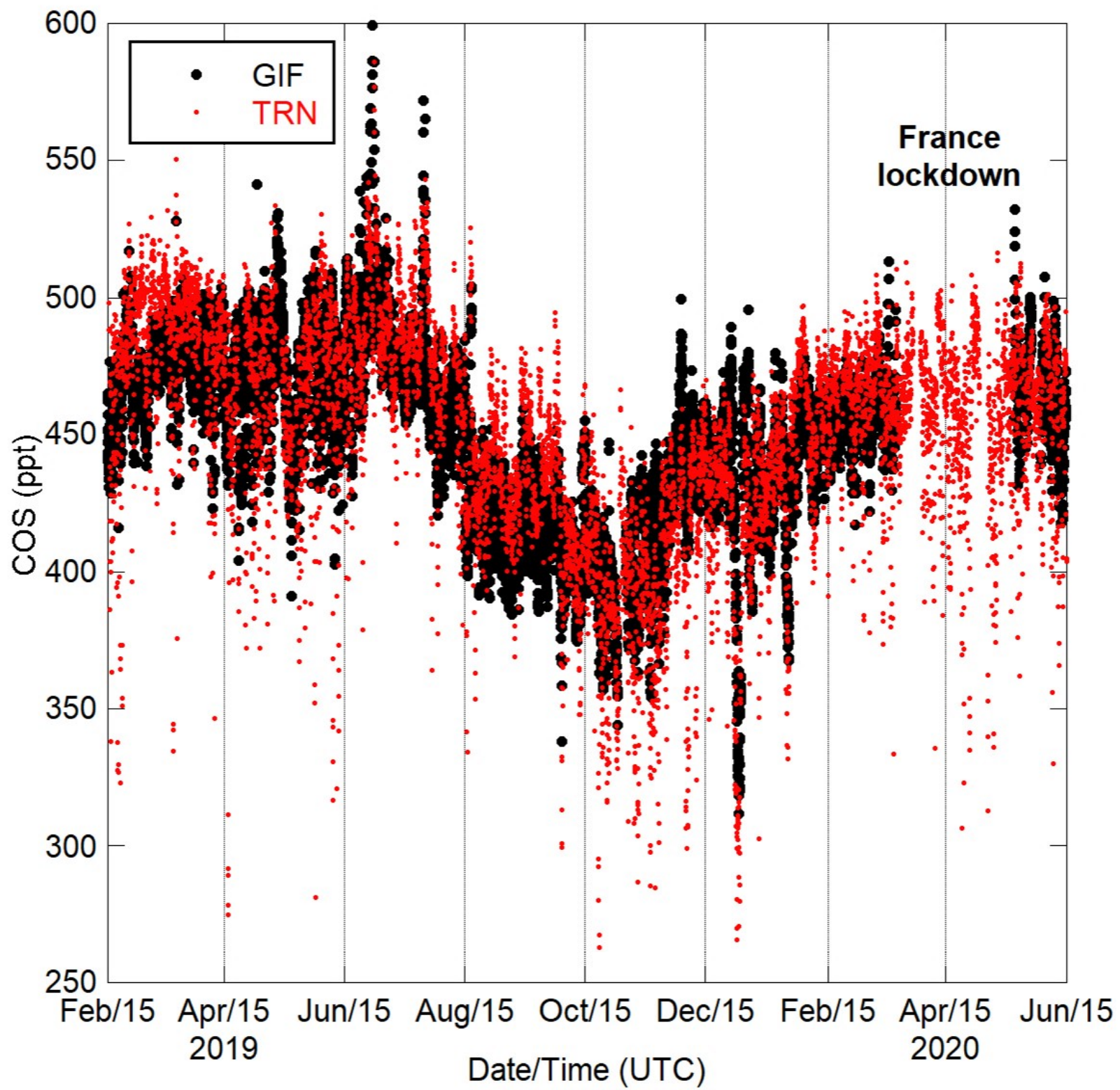

Supplement: S4 Fig — The mini-QCL remained operative at TRN during the French lockdown while GC measurements at GIF were stopped for about two months. (PDF) [file pone.0278584.s004.pdf]

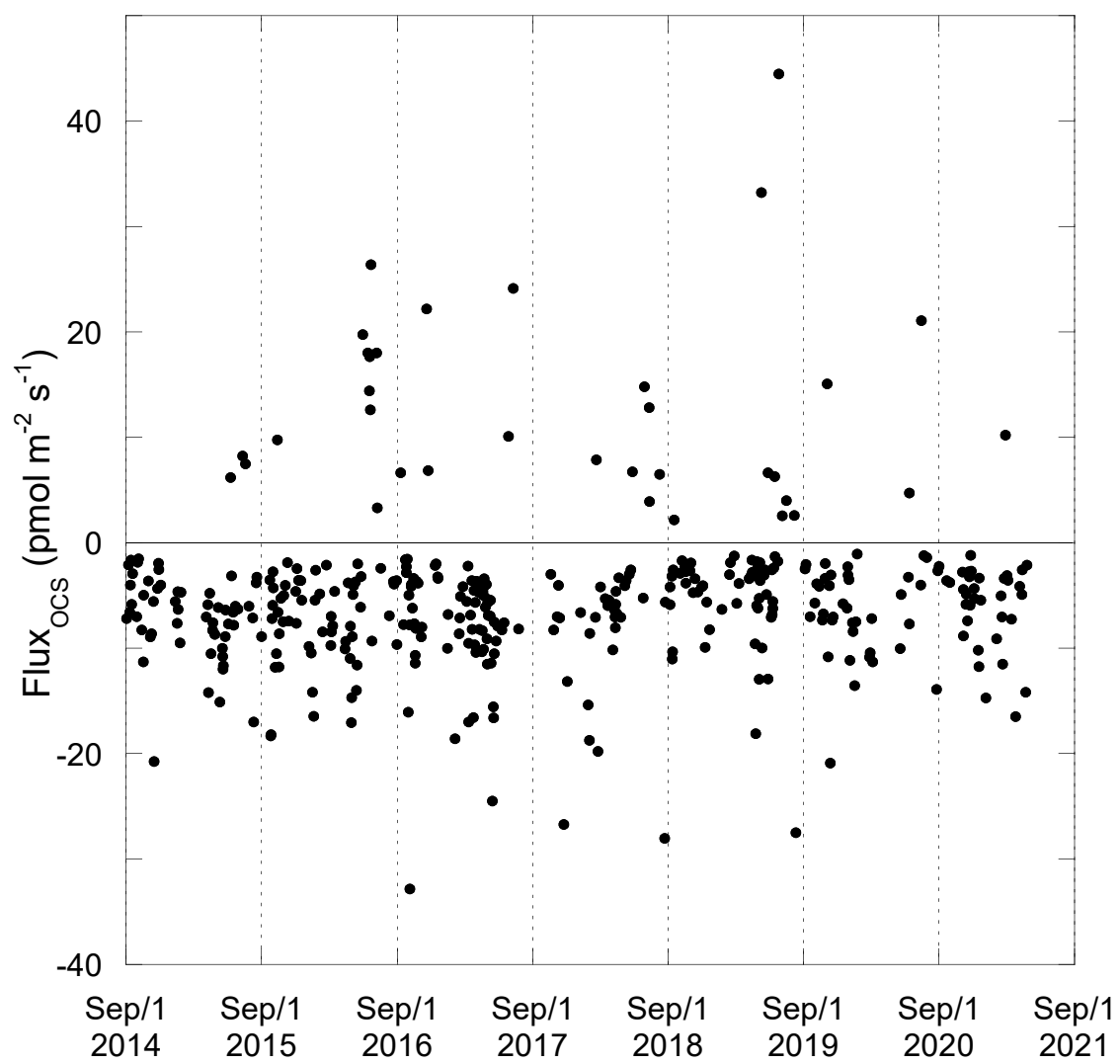

Supplement: S5 Fig — These are nocturnal COS (OCS) fluxes obtained by the Radon Tracer Method. (PDF) [file pone.0278584.s005.pdf]

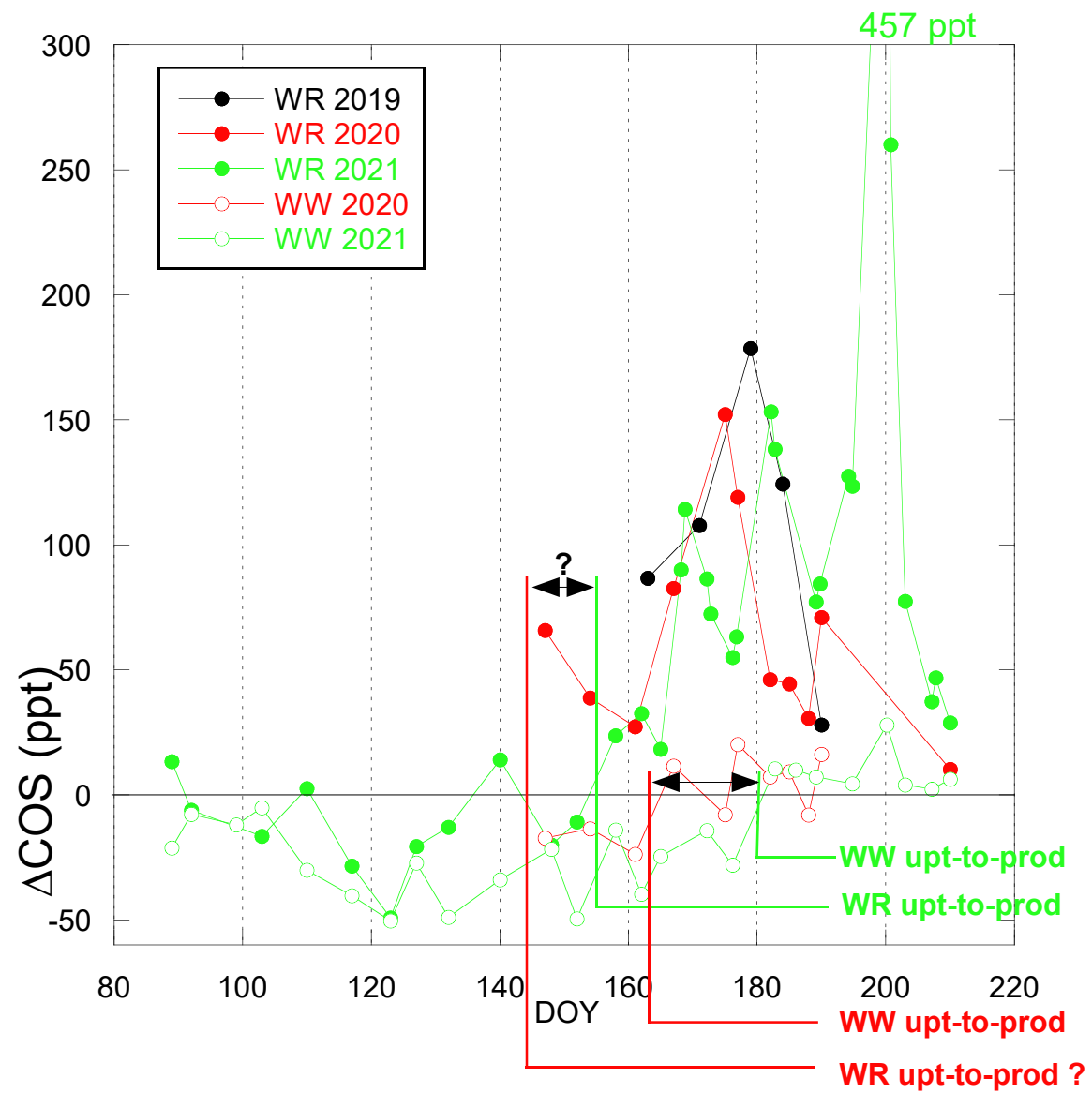

Supplement: S6 Fig — The difference in COS concentrations measured downwind and upwind of selected plots is plotted against day of year (DOY). Measurements were carried out in the morning between 9:00 and 11:00 (local time), during no rainy days and roughly in similar meteorological conditions according to wind speed (2 < WS < 14 km h-1, mean = 6 km h-1, SD = 3.6 km h-1). In 2021, the survey of crops was interrupted before harvest, whereas in 2020, the last sample was collected after harvest. The lag in 2021 of the shift from net uptake to net production (upt-to-prod) for either WW or WR is depicted by an horizontal double arrow. Rapeseed growth stages—year 2021: DOY<110, inflorescence emergence and elongation; >110–140, flowering; >140–160, development of fruit; >160–190, ripening; >190–210, senescence. We have zoomed in the May-to-July period in Fig 4D. (PDF) [file pone.0278584.s006.pdf]
