# Supplementary material for: Carbonyl sulfide (COS) emissions in two agroecosystems in central France
Source: PLoS One. 2022 Dec 6;17(12):e0278584. doi: 10.1371/journal.pone.0278584 (PMC9725148; doi:10.1371/journal.pone.0278584)
Supplement: S7 Fig — The SWI is a soil moisture index documented in the scientific literature. It represents, over a depth of about two meters, the state of the water reserve of the soil in relation to the useful reserve (water available for plant nutrition). Plots downloaded from https://donneespubliques.meteofrance.fr/?fond=produit&id_produit=129&id_rubrique=29. First, we selected Bulletin climatique mensuel régional (à partir de janvier 2020), then Ile-de-France region from the drop-down menu, then we downloaded reports for the months of July 2020 and July 2021, then compared graphs entitled “Indice d’humidité des sols” in page 4 of 5). Upper panel: March 1st to July 31st, 2020. Lower panel: March 1st to July 31st, 2021. Refer only to the purple curves. (PDF) [file pone.0278584.s007.pdf]

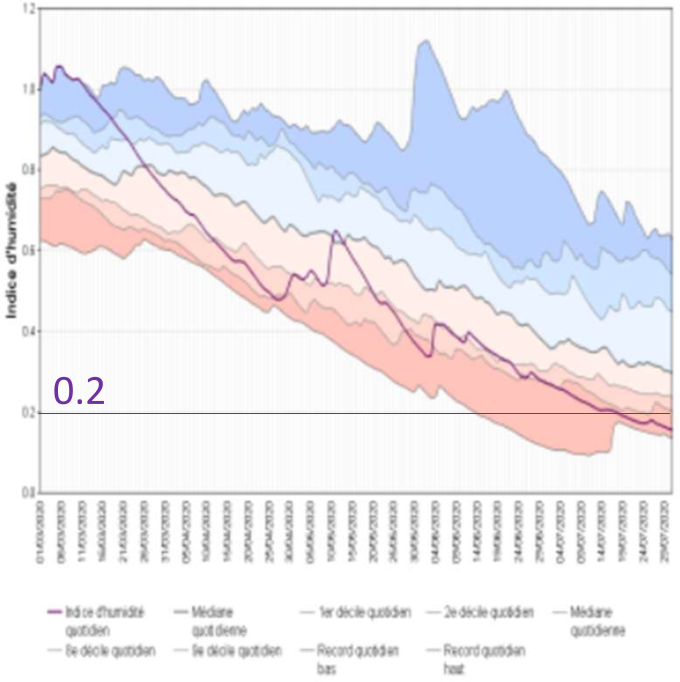

Indice d'humidité des sols
1er mars - 31 juillet 2020

Indice d'humidité des sols agrégé

Ile-de-France

1er mars 2021 au 31 juillet 2021

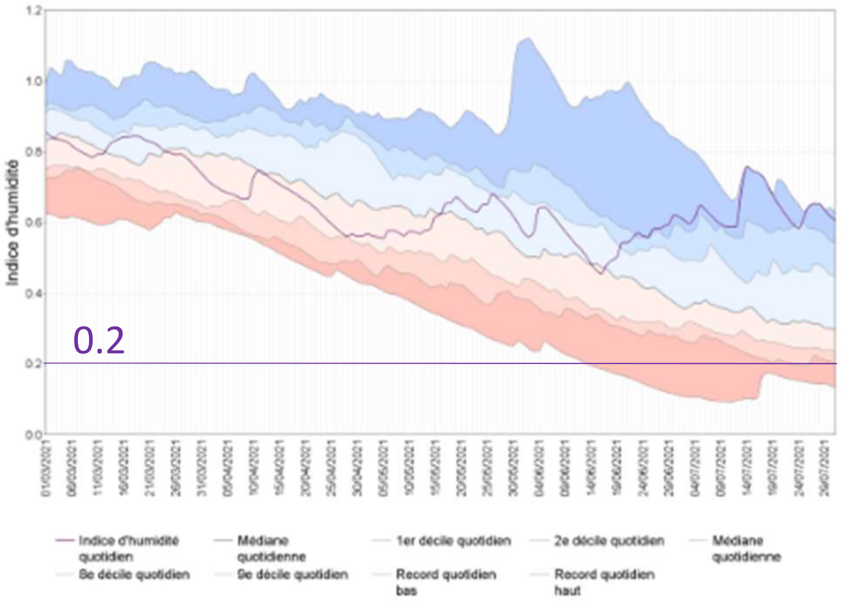

Edité le : 24/08/2021 - Produit élaboré avec les données disponibles du : 24/08/2021 à 14:57 UTC
