# Supplementary material for: Carbonyl sulfide (COS) emissions in two agroecosystems in central France
Source: PLoS One. 2022 Dec 6;17(12):e0278584. doi: 10.1371/journal.pone.0278584 (PMC9725148; doi:10.1371/journal.pone.0278584)
Supplement: Attachment — Submitted filename: Response to Reviewers.docx [file pone.0278584.s008.docx]

Reviewer #1: The study by Belviso et al. aims to examine the source and sink dynamics of carbonyl sulfide over wheat and rapeseed fields in the Paris region. The authors used two independent methods to infer nighttime COS fluxes (the radon tracer method and the gradient method) and the horizontal gradient method to qualitatively assess daytime COS fluxes. Although these methods each have their own gaps and the authors did not provide an ecosystem budget, daytime COS enhancements at downwind locations seem to provide evidence that the rapeseed field was a strong COS source during ripening and senescence. In my opinion, this is the main novelty and probably the most solid part of the work. However, the interpretation of the results suffers from several conceptual confusions and methodological weaknesses. There are three major issues that need to be addressed and some room for improvement in other aspects.

**We thank the reviewer for its rather positive appraisal of the manuscript, for the high quality of the review process and for its constructive comments.**

**Our study also aims to evaluate against field observations new model simulations of plant COS uptake and soil COS uptake and production, computed with mechanistic-based models recently implemented in the ORCHIDEE land surface model. This is an important aspect of our approach that we will defend in the following.**

First, the authors attribute COS emissions to rapeseed plants throughout the discussion. While the observed COS enhancement downwind of the rapeseed field (Fig. 5D) indicates the presence of a COS source in this ecosystem, it cannot tell whether this source is in the plants or the soil. As the authors are aware, both rapeseed plants (Bloem et al., 2012) and agricultural soils (Maseyk et al., 2014; Whelan et al., 2015) have been found capable of emitting COS. Without in situ soil flux or leaf flux measurements, there is no reason to exclude the possibility of a COS source in soils.

**Indeed, as discussed in the manuscript, both rapeseed plants (Bloem et al., 2012) and agricultural soils (Maseyk et al., 2014; Whelan et al., 2015) have been found capable of emitting COS. However, wheat plants have also been found capable of emitting COS during the senescence phase (Maseyk et al., 2014, see Fig. 1, panel C, reproduced hereafter,** [**https://doi.org/10.1073/pnas.1319132111**](https://doi.org/10.1073/pnas.1319132111)**). During senescence (doy 135–145) and after harvest (after doy 145), soil fluxes shifted to emissions only, and the source strength and amplitude of the diurnal variation increased (Maseyk et al. Fig. 1B). The difference between the green points (vegetation and soil fluxes) and the gray shading (soil emission only) was much higher during the senescence phase than after harvest (Maseyk et al. Fig. 1C). Note that brown wheat grain heads were also found to be producing COS. Conversely, during our survey dated 2020 (Fig. 4D) the COS horizontal gradient (ΔCOS) was markedly higher during the senescence phase than after harvest (i.e., 25-150 ppt vs. 10 ppt, respectively). This is the first field evidence that allows us to exclude the possibility that the soil dominates ecosystem fluxes during the senescence phase at GIF.**

**The second reason to exclude the possibility that the soil COS fluxes explain the differences between COS budgets over wheat and rapeseed plots is related to the spatial dimension of the survey areas and to the agricultural practices in the GIF area. In the studied area, cultivation is indeed restricted to the level top positions of the Saclay plateau and to deep, freely-drained and silty Luvisols developed in quaternary aeolian loess deposits (Choquet et al., 2021). All the cultivated plots sampled in our study area, which are less than 500 meters apart (cf. Fig 1A), therefore show very similar soils. Moreover, rotation of cultures is a general feature of agricultural practices in the GIF area. For example, at the plot within the ellipse displayed in Fig. 1A where winter rapeseed (WR) was grown in 2019, winter wheat (WW) was grown in 2018, maize (M) in 2017, M and WW in 2016, and M and spring rapeseed (SR) in 2015. We here rely on the annual EU Common Agricultural Policy maps (2015-2021) and data extracted from these maps are shown in the following table.**

| **Crop surveys** | **2021** | **2020** | **2019** | **2018** | **2017** | **2016** | **2015** |
| --- | --- | --- | --- | --- | --- | --- | --- |
| **WR plot sampled in 2020 and 2021** | **WR** | **WR** | **M** | **WW** | **WW** | **SR** | **WW** |
| **WR plot sampled in 2019** |  |  | **WR** | **WW** | **M** | **M & WW** | **M & SR** |
| **WW sampled in 2021** | **WW** | **WW** | **WW** | **M** | **WW** | **SR** | **WW** |
| **WW sampled in 2020** |  | **WW** | **WW** | **M** | **WW** | **SR** | **WW** |

**In other words, rapeseed is not grown as a monoculture in the GIF area which could have allowed soil to develop specific COS production capacities.**

**Finally, it is unlikely that soil fertilization with sulfur would promote COS production because only the wheat field, which displays almost no COS net production, was amended with sulfur in 2021.**

**References:**

**Choquet, P., B. Gabrielle, M. Chalhoub, J. Michelin, O. Sauzet, O. Scammacca, P. Garnier, P.C. Baveye, and D. Montagne (2021), Comparison of empirical and process-based modelling to quantify soil-supported ecosystem services on the Saclay plateau (France), Ecosystem Services, 50, https://doi.org/10.1016/j.ecoser.2021.101332.**

Second, the authors interpret the difference between fluxes simulated by the ORCHIDEE model and derived from COS concentration observations simplistically as “additional ecosystem COS flux” (Fig. 5B). They then interpreted the exceptionally high difference between observed and modelled fluxes in May–July as evidence for a missing COS source (Fig. 5C). This is fraught with issues because many factors lead to model–data discrepancies. For example, the model’s default set of plant physiological parameters determining COS fluxes may differ from the parameters measured in the field. This is because land surface models typically represent plants at the functional type level, not at the species level. When models do represent major crop species, they may use a general set of parameters and not have the granularity to finely resolve crop varieties actually grown in the field, unless they are customised to do so. Because simulated nighttime COS fluxes critically depend on nighttime stomatal conductance (Kooijmans et al., 2017), model–data discrepancies could also be attributed to misrepresented nighttime stomatal conductance (which we know to be the case for many models, e.g., Kooijmans et al., 2021). In addition, the large discrepancy in May–July could be due to misrepresented soil moisture limitation on stomatal conductance. There is insufficient evidence to pin this discrepancy on a missing COS source.

**The reviewer is right. However, the stomatal conductance values used in ORCHIDEE were compared to the one found in Lombardozzi et al. (2017) in Table A3 of Maignan et al. (2021) reproduced below. The minimum stomatal conductances in ORCHIDEE are lower than the ones from Lombardozzi et al. (2017). If we adapted the residual stomatal conductance in ORCHIDEE based on Lombardozzi et al. (2017), it would tend to increase vegetation COS uptake at night and, as a consequence, higher “additional ecosystem COS fluxes” will be expected when the reviewer expected lower ones.**

**We are aware that land surface models simulate COS exchanges between the atmosphere and the vegetation when the later displays significant stomatal conductance and photosynthetic activity. In fact, we simulate vegetation COS uptake even when there is no photosynthetic activity, as during nighttime when stomatal closure is incomplete. During nighttime, we implemented a minimum stomatal conductance using the residual stomatal conductance when irradiance approaches zero, multiplied by a factor representing water stress. When plants reach the ripening and senescence phases the leaves progressively lose these capacities. That is why it is argued that although “the model’s default set of plant physiological parameters determining COS fluxes may differ from the parameters measured in the field”, this is no more a major issue when plants reach the ripening and senescence phases. According to the role of soils in the budget of COS, the last version of the ORCHIDEE-COS model (Abadie et al., 2022) relies on our current understanding of the production and removal processes taking place in the soil. A nocturnal net production of COS was simulated by the ORCHIDEE model essentially during a heatwave which lasted a few days in July 2019 at the TRN site. At GIF, this heatwave only resulted in a reduction of the nocturnal net uptake of COS (Fig. 5A). However, the results of the following sensitivity test, where simulations only differ according to the soil texture, show that lower COS soil production rates are reached when using Zobler's assessment of FAO Soil Units (Zobler, 1986) instead of FAO-USDA (This work and Abadie et al. (2022)) during summers and the summer heatwave dated 2019. Therefore, the current version of the ORCHIDEE/COS model based on ORCHIDEE minimum conductances (Table A3) and soil texture from USDA already tends to reduce nighttime ecosystem COS fluxes so that lower “additional ecosystem COS fluxes” need to be invoked.**

Third, given the patchy nature of the land use map (Fig. 1), it is unclear how the authors achieved a clear separation of wheat vs. rapeseed influences on the downwind COS enhancements. Spatial heterogeneity presents a bigger problem for the TRN site. Because measurements at 180 m cover a greater footprint area than measurements at 5 m, the fluxes calculated from concentration gradients between the two levels are not specific to a single land use type.

**The surveys at the TRN site are not meant to achieve neither a “clear separation of wheat vs. rapeseed influences” nor to “calculate fluxes from concentration gradients”. Our approach aims to assess monthly changes in nocturnal COS net fluxes from the survey of relative vertical gradients of COS (Grad_COS_/Grad_CO2_) in a rural area where many crop varieties are grown (Fig. 1B). It is likely that measurements at 180 m cover a greater footprint area than measurements at 5m. However, we will demonstrate in the following that, at the TRN site in the summers of 2019 and 2020, the 5 m and 180 m inlet heights remain within the nocturnal boundary layer (NBL) throughout the night. The diurnal and seasonal variability of the atmospheric boundary layer (ABL) depth (zi) at the TRN site has been extensively investigated by Pal et al. (2015) from lidar measurements, vertical profiles of CO2 at 5, 50, 100 and 180 m along the 207 m tall tower, and 222Rn measurements at 180 m. The authors showed that, in 33.9% of cases, zi was below the top of the tower during the summer (JJA) nights of 2011 ([Pal et al., cf. Fig. 5C reproduced below).**

**During the summer period, scenario A (see their Fig. A1 reproduced below), which is the scenario envisaged by the reviewer, is less likely than scenario B where the 5 and 180 m inlets remain within the NBL.**

**The following plots, available from the ICOS database for the years 2019 and 2020, show that in May, June, July and August (central row), the nocturnal CO_2_ monthly means at 180 m height remain 1.7 to 7.7 ppm (in 2019) and 2.7 to 6.2 ppm (in 2020) higher during the night (black curve, strong stratification) than during the day (blue curve, strong vertical mixing). It indicates (1) that the 180 m inlet does not sample air from the residual layer (RL) by night, in support of scenario B, and (2) that it is likely that the concentration gradients between the two levels are specific to a single land use type. The manuscript has been updated accordingly.**

**References:**

**Pal, S., M. Lopez, M. Schmidt, M. Ramonet, F. Gibert, I. Xueref-Remy, and P. Ciais (2015), Investigation of the atmospheric boundary layer depth variability and its impact on the ^222^Rn concentration at a rural site in France, J. Geophys. Res. Atmos., 120, 623–643, doi:10.1002/2014JD022322.**

Given these weaknesses, it seems more fruitful for the manuscript to focus on results of relatively high confidence, such as large COS emissions from the rapeseed field. If the authors want to make the claim of a large COS source in rapeseed plants, they need to measure soil or leaf COS fluxes, not relying on atmospheric COS concentrations to resolve what cannot be resolved from a top-down point of view. They would also need to calibrate parameters of the ORCHIDEE model against those measured on wheat and rapeseed plants in the field, before suggesting a missing COS source (but this would not be needed if a COS source is directly observed in the field). Alternatively, the interpretation must be revised to reflect alternative explanations.

**We revised the interpretation to reflect alternative explanations, yet less likely as the one we put forward in the manuscript and as discussed previously in this document.**

The presentation of the manuscript could be streamlined to benefit readers who may not be well-versed in carbonyl sulfide research or flux quantification methods. Rather than confronting the reader with the current uncertainty in COS budgets, the introduction could start with a brief statement on why we care about COS and why there is a need to examine its sources and sinks in croplands. It would be better to list the description of the three flux quantification methods—horizontal gradient method for daytime fluxes at GIF, radon tracer method for nighttime fluxes at GIF, and vertical gradient method at TRN—under separate headings to avoid potential confusion.

**First, please refer to our reply to the specific comment L174–175 where it is stated that the aerodynamic gradient method (e.g., Rastogi et al. (2018)) and the vertical gradient method applied at TRN are distinct approaches, and that the former is not suitable at TRN. The beginning of the introduction has been rewritten as follows:**

**“The uptake of atmospheric carbonyl sulfide (COS) through stomata in the plant leaves and subsequent irreversible hydrolysis by enzymatic reaction with carbonic anhydrase (FCOSveg.) is the largest sink in the global budget of this compound, yet estimates differ by as much as a factor of 5.6 as discussed in a recent review [1]. However, if estimates of FCOSveg. based on net primary production (NPP) scaling are excluded, since it is now known that FCOSveg. has to be scaled rather to gross primary production (GPP) than NPP, the rest of the estimates based on GPP scaling or mechanistic models vary by only a factor of two [1]. A larger uncertainty is…”**

Lastly, I commend the authors for making the data openly available with no strings attached. Please consider putting the data set in long-term archives (for example, Zenodo) so that it can be easily searched, accessed, and cited.

**The GIF time series of COS atmospheric mixing ratios and fluxes, and the vertical profiles at TRN are now also available in a long-term archive (**<https://data.ipsl.fr/catalog/srv/eng/catalog.search#/metadata/6800b065-dcec-4006-ada5-b5f62a4bb832>**) and** <https://doi.org/10.14768/6800b065-dcec-4006-ada5-b5f62a4bb832>.

Specific comments

L23: “exchange fluxes” -> “fluxes”

**Done.**

L23–24: “simulated by vegetation and soil models, implemented in the ORCHIDEE land surface model” -> “simulated by the ORCHIDEE land surface model”

**This point has been a matter of debate within our group. We have reached the agreement that COS fluxes simulated by vegetation and soil component models were implemented in the ORCHIDEE land surface model.**

L32: Readers would not have known what the “profile vs. model” approach means at this point. Explain the approach and to which modelled variables the profile is compared.

**The “profile vs. model”, “RTM vs. model” and “crop based” approaches evaluate simulated fluxes against field observations at two agroecosystems in central France. It is now stated in the abstract that “The dynamics of a biogenic process yet unaccounted for by this model, i.e., COS emissions from croplands, was examined in the context of three independent and complementary approaches, First, during the growing seasons of 2019 and 2020, monthly variations in the nighttime ratio of vertical mole fraction gradients of COS and carbon dioxide measured between 5 and 180 m height (GradCOS/GradCO2), a proxy of the ratio of their respective nocturnal net fluxes, were monitored at a rural tall tower site near Orléans (i.e., a “profile vs. model” approach).”**

L34: “were used for that same purpose” - Unclear from the context. Specify the purpose.

**The abstract now reads: “Second, field observations of COS nocturnal fluxes, obtained by the Radon Tracer Method (RTM) at a sub-urban site near Paris, were used for that same purpose (i.e., a “RTM vs. model” approach of unaccounted biogenic emissions).”**

L39–40: “the nocturnal net COS uptake gradually weakens” - The weakening of nocturnal net COS uptake does not imply the existence of a source. It could be due to the reduction of nocturnal stomatal conductance in response to increased atmospheric and soil water stress in the late growing season.

**Please refer to our answer to the reviewer’s second general comment.**

L43–44: The downwind enhancement of COS concentration over the wheat field is too small to indicate a robust COS source. How does the difference compare to uncertainty from measurement and atmospheric transport?

**You are right. The text has been updated accordingly as follows: “Our “crop based” comparative approach demonstrates that rapeseed crops shift from COS uptake to emission in early summer during the late stages of growth (ripening and senescence) while wheat crops uptake capacities lower markedly.”**

L53–54: “yet estimates differ by as much as a factor of 5.6 as the recent review of [1] shows” - If estimates based on NPP scaling (which we now know is incorrect understanding) are excluded, the rest of the estimates based on GPP scaling or mechanistic models vary by a factor of two only.

**You are right. The introduction has been updated accordingly: “The uptake of atmospheric carbonyl sulfide (COS) through stomata in the plant leaves and subsequent irreversible hydrolysis by enzymatic reaction with carbonic anhydrase (FCOSveg.) is the largest sink in the global budget of this compound, yet estimates differ by as much as a factor of 5.6 as discussed in a recent review [1]. However, if estimates of FCOSveg. based on net primary production (NPP) scaling are excluded, since it is now known that FCOSveg. has to be scaled rather to gross primary production (GPP) than NPP, the rest of the estimates based on GPP scaling or mechanistic models vary by only a factor of two [1].” Thank you for that very helpful suggestion.**

L66–67: “vascular plants could play also a role in COS production” - I would add that this happens for a limited number of species (Geng et al., 2006), during senescence (Maseyk et al., 2014), or with fungal infections (e.g., Bloem et al., 2012), lest readers think it is a common behaviour.

**The text now reads: “Although laboratory and field observations have shown in rare cases that vascular plants could play also a role in COS production [7-9], the processes by which plants emit this gas have yet to be considered in global modeling studies.”**

L103–107: This is a convoluted sentence. Simplify it.

+

L105–107: “the fields over which the order of magnitude of COS fluxes were assessed indirectly from downwind minus upwind horizontal concentration gradients along the wind direction” - This seems to belong to the measurement methods, not site description.

+

L107–119: It would be better to separate the description of crop planting and harvesting from that of the monitoring station.

**The different sentences and paragraphs have been reorganized in depth as follows:**

**Experimental sites / Applied flux quantification methods**

**Atmospheric boundary layer mixing ratios were monitored at two sites, Gif-sur-Yvette (GIF) and Trainou (TRN tall tower), both in central France (Fig 1).**

**TRN site: nocturnal vertical gradients**

**The Trainou 180-m tall tower atmospheric observatory is located about 80 km south of GIF (Fig 1). A description of the TRN site is available online at https://icos-atc.lsce.ipsl.fr/panelboard/TRN. At the TRN tall tower, atmospheric COS was measured with the Aerodyne Research quantum cascade laser (mini-QCL) formerly deployed at the SAC station [14]. The sampling lines, made of Synflex tubing, collected air at 4 heights (5 m, 50 m, 100 m and 180 m) sequentially, the total sequence lasting for 80 minutes. Because the TRN station is operated in the framework of ICOS European Infrastructure [15], the mini-QCL was synchronized with an ICOS analyzer measuring CO2, CH4, CO and H2O (Picarro Model G2401). A proxy of the ratio of nocturnal ecosystem fluxes (FCOS/FCO2) was calculated from nighttime vertical gradients of COS and CO2 (GradCOS/GradCO2 from data collected at 5 m and 180 m height), assuming similarity of COS fluxes and gradients to CO2 flux-gradients. Data used to calculate the linear regression slope between GradCOS and GradCO2, were selected according to time of day as follows: [21; 4[ (UTC) in spring and summer since we only aimed at examining the capacity of agricultural ecosystems in exchanging COS with the atmosphere. The linear regression slope between GradCOS and GradCO2 is forced through zero because vertical gradients tend to zero during windy nights. The relative vertical gradients of COS measured at TRN between 5 and 180 m can be used to assess local exchange rates provided that the highest inlet height remains within the nocturnal boundary layer (NBL) throughout the night. This necessary condition was achieved during the months of May through August of 2019 and 2020 as shown later from an analysis of the diurnal variations in monthly mean CO2 profiles. Emission and uptake rates of COS from nearby crops were not investigated from horizontal mole fraction gradients there.**

**GIF site: RTM method and horizontal concentration gradients**

**The GIF monitoring station is located at LSCE, l’Orme des Merisiers, about 2 km south of the Saclay station (SAC) part of the Integrated Carbon Observation System (ICOS) network (https://icos-atc.lsce.ipsl.fr/panelboard/SAC), and next to the village of Saint Aubin. For more information about land cover classification, see Fig 1 of [14] and the EU Common Agricultural Policy (CAP) maps shown in Fig 1.**

**At GIF, atmospheric COS was measured at 7 m, on line every hour using an automated sampling system (Entech P7100) coupled to a gas chromatograph (GC, Varian 3800) [14,16]. The GIF time series currently covers seven years (from August 2014 to September 2021). COS fluxes were computed with RTM as by [14], an approach assuming comparably homogeneous spatial distributions of the soil 222Rn source and the ecosystem COS sink/source. COS fluxes are computed as the product of COS/222Rn slopes during nighttime inversion and 222Rn local exhalation rates. The latter (i.e., 52 Bq m-2 h-1 with a seasonal cycle amplitude of +25% in summer and -25% in winter driven by soil moisture) was taken from [17]. The RTM allowed us to make 400 determinations of nocturnal fluxes of COS throughout the 2014-2021 period as detailed later.**

**The small ellipse on the left panel of Fig. 1 delineates the fields from which COS fluxes were assessed indirectly from downwind minus upwind horizontal concentration gradients along the wind direction (WD). First, wind speed (WS) and WD data from a weather station located on the LSCE roof top (Vaisala model WXT530) was used to approximate local meteorology. In the field, we used the facilities of a local meteorological station belonging to the Institut National de la Recherche Agronomique (INRA), equipped with a wind vane providing a continuous visualization at the local scale of the WD. WS data from the INRA weather station was downloaded later. Horizontal COS concentration gradients were determined discontinuously in spring-summer of 2019, 2020 and 2021. They were documented between 9:00 and 11:00 AM (local time), during non-rainy days, from flask-air samples collected in pairs each upwind and downwind (2 < WS < 14 km h-1, mean = 6 km h-1, SD = 3.6 km h-1) of selected crops fields surrounded by the ellipse in Fig 1A. Although the total duration of the crop surveys was 2 hr, each horizontal gradient was documented within 40 minutes. We ensured that the concentration gradient was not affected by the residual nocturnal boundary layer by comparing samples collected on an hourly basis at 7 m (GIF time series) with upwind flask samples collected at canopy height. The sampling device has been described by [18]. Once filled with sample air, flasks were transferred within hours to the laboratory nearby and analyzed for COS with the Entech/Varian instruments described below. Precision for GC measurements of flask-air samples is reported as box plots in Fig 2.**

**During May to July of 2021, 29 pairs of flasks were collected and each flask was analyzed twice consecutively for COS. The average difference between duplicated analyses was 9.3 ± 7.2 ppt (median = 7.5 ppt, interquartile range (IQR) = 4.5 – 12.5 ppt, n=58). The average difference between flasks of the same pair, assessed as the median of flask 1 minus that of flask 2, was 5.5 ± 4.5 ppt (median = 4.9 ppt, IQR = 2.2 – 8.0 ppt, n=29). In 90% of the analyses, the difference between flasks of the same pair was less than 14 ppt.**

**Our study of uptake/emission from the rapeseed and wheat fields covers a measurement period from March 30th to July 29th, 2021, encompassing most important growth stages, i.e., stem elongation/extension, flowering, ripening and senescence. Winter rapeseed (Blackbuzz variety) was sown on September 5th, 2020 and harvested August 6th, 2021. A mixture of four varieties of winter wheat (Renan, Gwenn, LG Absalon, Chevignon) was sown on October 31st, 2020 and harvested August 14th, 2021. Agricultural treatments, including herbicides, insecticides and nitrogen fertilizers, were applied to each crop in autumn and winter. The wheat field was fertilized with sulfur in early March 2021. Wheat and rapeseed yields were 6.8 and 3.7 t ha-1 in 2021, respectively. COS exchanges could not be investigated in 2020 as in 2021 because of the French COVID-19 lockdown. All the cultivated plots sampled in our study area, which are less than 500 meters apart (Fig 1A), show very similar soils and the rotation of cultures is a general feature of agricultural practices in the GIF area.**

L116–117: Was the rapeseed field fertilised with sulfur as well?

**No, it was not.**

L128: “They were documented in the morning between 9:00 and 11:00 (local time)” - How would you ensure that the concentration gradient was not affected by the residual nocturnal boundary layer?

**The text has been updated accordingly: “Although the total duration of the crop surveys was 2 hr, each horizontal gradient was documented within 40 minutes. We ensured that the concentration gradient was not affected by the residual nocturnal boundary layer by comparing samples collected on an hourly basis at 7 m (GIF time series) with upwind flask samples collected at canopy height.”**

L129: “from flask-air samples collected in pairs each upwind and downwind” - Did you measure the wind speed and direction? Was there any change in the wind regime during sample collection? Did the downwind sampling location receive influences from places other than the upwind sampling location (i.e., how were the back-trajectories like?).

**We measured the wind speed (WS) and direction (WD) from a weather station located on the LSCE roof top (Vaisala model WXT530) before leaving the LSCE building in order to approximate local meteorology. In the field, we used the facilities of a local meteorological station belonging to the Institut National de la Recherche Agronomique (INRA), equipped with a wind vane providing a continuous visualization at the local scale of the WD (see the picture below). WS data from the INRA weather station was downloaded later.**

L174–175: This method implicitly assumes that COS and CO2 have the same aerodynamic conductance. This assumption may not hold because we know that COS and CO2 have different molecular diffusivities in the air. Please consult Rastogi et al. (2018) J. Geophys. Res. Biogeosci. for the calculation of aerodynamic conductance and a rigorous application of the gradient method. You may also want to consider the storage fluxes depending on how fast the concentration gradients change in time (see Eq. 2 in Rastogi et al., 2018).

**The diurnal and vertical variations of COS at the Wind River Experimental Forest (WREF), located within the Gifford Pinchot National Forest in southwest Washington state (USA, copied from Rastogi et al. (2018) and shown below) and at TRN share very little in common. At WREF, COS maintained a stronger gradient at all heights throughout the daylight hours than during the nighttime ones. Rastogi et al. (2018) stated: “Canopy flushing of higher concentration atmospheric OCS results in a daytime increase in measured mixing ratios throughout the canopy” and “at Wind River, storage fluxes of OCS are substantially larger than those observed in other ecosystems (e.g., Kooijmans et al., 2017). This is in part due to the significantly larger volume of the canopy airspace of the tall old growth forest (the tallest Douglas fir trees are between 50 and 60 m), but also due to the site’s proximity to the ocean (major source of atmospheric OCS)”.**

**In contrast, COS vertical gradients are essentially observed during the nighttime hours at TRN (Fig. S3) where the landscape of the region is flat, surrounded by agricultural fields (50%), forests (30%, the typical canopy height is around 15 m agl), and the distance to the nearest coast line is about 320 km (Pal et al., 2015). The major conceptual difference between Rastogi’s approach of biogenic fluxes and ours is that the former investigated the net ecosystem exchange of COS (NEE_COS_ = F_COS_ + S_COS_) from canopy fluxes (F_COS_), measured by the aerodynamic gradient method at the top of the canopy (i.e. between 60 and 70 m), and storage fluxes (S_COS_) which were estimated as the integral of concentration changes over height up to the height of the profile system (i.e., 70 m). Note that in Kooijmans et al. (2017) F_COS_ was measured by the eddy-covariance technique. It is not possible to apply the approach of Rastogi or that of Kooijmans at the TRN site because the 50, 100 and 180 m inlets are located far higher than the typical canopy height and the 5 m inlet much too low. In fact, the TRN tall tower is not designed to specifically investigate biogenic fluxes neither by the aerodynamic gradient method nor by the eddy-covariance technique. The relative vertical gradients of COS measured at TRN between 5 and 180 m can be used to assess local exchange rates provided that the highest inlet height remains within the nocturnal boundary layer (NBL) during the entire night as demonstrated previously.**

L226–227: “the site is a net sink” - Only at night. The radon tracer method does tell anything about daytime fluxes.

**Correct.**

L229–230: “More precisely, emission episodes were systematically recorded during the months of May through July over six consecutive years.” - What drives nighttime emissions? Did these episodes correlate with high temperatures or high VPD?

**The ORCHIDEE land surface model is forced by 0.25°x0.25° hourly reanalysis from the fifth generation of meteorological analysis of the European Centre for Medium-Range Weather Forecast. It is from the comparison between simulations and observations that one can investigate the role of physical parameters such as temperature and/or VPD.** **Since the model fails to reproduce the seasonality and magnitude of the nighttime emissions of COS, we doubt that the later could be linearly correlated with physical parameters such as temperature or VPD. Nevertheless, during the major heatwave that stroke the Ile-de-France region in August 2020 (from August 6^th^ to 9^th^), where recorded temperatures were higher than 30°C and the relative humidity lower than 25% (see the following ICOS data product), the RTM method did not report any emission episode (Fig. 4A).**

L234–243: This paragraph seems to belong to the methods.

**We estimate that this paragraph rather belongs to the results than to the methods because it is a preliminary illustration of the approach aiming at assessing monthly changes in nocturnal net exchange rates from the survey of relative vertical gradients of COS (GradCOS / GradCO2). We start with the 2020 observations because they are the shortest (only two month duration).**

L256–257: “This suggests that the efficiency of the ecosystem to take up COS from the atmosphere by night significantly lowers as the growing season proceeds.” - As stated earlier, this change could result from a reduction in nocturnal stomatal conductance and does not necessarily indicate the presence of a COS source.

**Please refer to our answer to the reviewer’s second general comment.**

L262–263: “a heatwave which lasted only for few days in July 2019 and resulted in enhanced soil production of COS” - Is there any observational evidence to indicate that COS is produced from the soil, not the vegetation?

**Not at the GIF and TRN sites. We are aware of the work of Cochavi et al. (2021) carried out in a Mediterranean citrus orchard. According to data gathered in their Fig. S8 and notes S1 (**[**https://nph.onlinelibrary.wiley.com/action/downloadSupplement?doi=10.1111%2Fnph.17247&file=nph17247-sup-0001-Supinfo.pdf**](https://nph.onlinelibrary.wiley.com/action/downloadSupplement?doi=10.1111%2Fnph.17247&file=nph17247-sup-0001-Supinfo.pdf)**), the COS flux partitioning using the temperature and moisture response equations of Yang et al. (2018) applied under the measured conditions of their study show that, during daytime extreme and intermediate heatwaves, the canopy and soil takes up atmospheric COS (canopy >> soil) while the litter produces COS. The net effect is COS uptake during the early morning hours (6-10 am) evolving into lower uptake later in the journey.**

**References:**

**Cochavi, A., M. Amer, R. Stern, F. Tatarinov, M. Migliavacca and D. Yakir (2021), Differential responses to two heatwave intensities in a Mediterranean citrus orchard are identified by combining measurements of fluorescence and carbonyl sulfide (COS) and CO2 uptake, New Phytol., 230 (4), 1394-1406, doi:10.1111/nph.17247.**

L294: As stated earlier, the discrepancy between simulated and observed fluxes is not enough evidence to suggest the presence of unaccounted COS emissions, because it could arise from poorly calibrated plant physiological or soil parameters.

**It is known that wheat is a source of COS during the senescence phase (Maseyk et al., 2014). As far as we are aware of, no comparison between simulated and observed fluxes in north-central Oklahoma (where Maseyk documented ecosystem and soil fluxes) has ever been conducted. We agree that the discrepancy between simulated and observed fluxes could arise from poorly calibrated plant physiological or soil parameters but, until this could be demonstrated there, the presence of unaccounted COS emissions from senescent plants or from brown wheat grain heads is likely. The discussion has been updated accordingly: “Maseyk et al. [8] were the first to report that wheat plots in Oklahoma in May-June 2012 act as a sink of COS during the early stages of growth (green plants), then shifted to COS release during the ripening and senescence phases. However, we are unaware of any comparison between simulated and observed fluxes in north-central Oklahoma, where Maseyk et al. [8] documented ecosystem and soil fluxes of COS. Hence, it is not known whether discrepancies exist between simulated and observed fluxes that could arise from poorly calibrated plant physiological or soil parameters rather than from COS emissions from senescent plants yet unaccounted for by models.”**

L302: At the ecosystem scale, “in situ” usually refers to eddy covariance flux measurements. Suggest removal.

**Done.**

L313: Are the numbers after the plus/minus sign one standard deviation?

**Yes.**

L317: “5.4 ± 11.2 ppt” - It does not seem that the COS enhancement over the wheat field is statistically significant. I suggest running a statistical test for the significance of the difference (e.g., a t-test for paired samples).

**We have run a** **Wilcoxon rank sign test for paired data to test for the significance of the difference between COS enhancements. The following P values have been added to the text.**

**2020: Δ Rapeseed-Wheat: DOY > 165: P=0.016**

**2021: Δ Rapeseed-Wheat: DOY ≤ 140: P=0.027**

**2021: Δ Rapeseed-Wheat: DOY > 165: P=0.002**

**2021: Δ Rapeseed-Wheat: DOY > 180: P=0.016**

L318–320: “Hence, it appears that the net production of COS by the rapeseed field lasted longer (15-25 day longer) and its intensity is about 14 times higher than that of the wheat plot.” - The downwind–upwind concentration enhancement depends on both COS emission rates and wind speed and direction. Without running an inversion or a dispersion model to infer the fluxes, concentration difference alone is not a good indicator of fluxes.

**Wind direction was documented in the field. The concentration gradients were systematically measured along the local wind direction. The plot dimensions are only of few ha. At this small spatial scale, the concentration difference is a relevant indicator of fluxes.**

L320–322: “The shift of wheat from net uptake to net production took place about 2 weeks later in 2021 than in 2020, likely because crops experienced a summer drought in 2020 and wetter conditions in 2021” - This seems an important point buried in the middle of the text. Could you test the influences of heatwaves or drought on emission episodes?

**Please refer to our answer to the reviewer’s second general comment.**

L322: “soil humidity index” - This needs a definition. Or, could you convert it to volumetric soil water content?

**The SWI (Soil Wetness Index) is a soil moisture index documented in the scientific literature. It represents, over a depth of about two meters, the state of the water reserve of the soil in relation to the useful reserve (water available for plant nutrition).** **Conversion of the regionally aggregated SWI index to local volumetric soil water content is not achievable because intermediate calculations are not available to us.**

L358–371: These sentences on VOC emissions do not seem relevant. We do not know if COS production is linked to the production of sulfur-free VOCs at the molecular and cellular levels. Suggest removal.

**“Exchanges of biogenic volatile organic compounds (BVOC) between plants and the atmosphere are likely to vary, in amount and composition, between different plant species but also for a single plant during its development. However, the effect of plant development stages, including senescence, on BVOC exchanges remains poorly investigated, especially in the case of crop plants” (Gonzaga Gomez et al., 2021). Using the same approach described in their paper dated 2019 (Gonzaga Gomez et al., 2019), the authors found that the overall BVOC emissions of winter wheat increased twofold during the senescence stage compared to the maturation stage (Gonzaga Gomez et al., 2021). This is qualitatively consistent with Maseyk et al. (2014)’s COS observations from wheat fields and ours over rapeseed crops, yet, as stated in our manuscript: “There is a lack of comparative studies of COS fluxes by wheat, rapeseed and potentially by other plants using dynamic chambers as for VOC fluxes”. This message could stimulate future work and can be understood as a perspective.**

**References:**

**Gonzaga Gomez L, Loubet B, Lafouge F, Ciuraru R, Buysse P, Duranda B, et al. Comparative study of biogenic volatile organic compounds fluxes by wheat, maize and rapeseed with dynamic chambers over a short period in northern. Atmospheric Environment. 2019; 214.** [**https://doi.org/10.1016/j.atmosenv.2019.116855**](https://doi.org/10.1016/j.atmosenv.2019.116855)

**Gonzaga Gomez L, Loubet B, Lafouge F, Ciuraru R, Bsaibes S, Kammer J, et al. Effect of senescence on biogenic volatile organic compound fluxes in wheat plants. Atmospheric Environment. 2021; 266.** [**https://doi.org/10.1016/j.atmosenv.2021.118665**](https://doi.org/10.1016/j.atmosenv.2021.118665)

L376–399: This paragraph feels disorganised. I would separate it into one paragraph on the biochemical mechanisms of COS production and another on the upscaled potential global budget of COS emissions from wheat and rapeseed fields.

**The paragraph has been reorganized accordingly as follows:**

**“COS is thought to be produced from isothiocyanates and thiocyanate ions (SCN-) which are secondary products of the action of the myrosinase enzyme onto a large variety of forms of glucosinolates, which themselves are naturally biosynthesized in plants of the “cabbage order”, Brassicales [29]. These authors also provided a list of about sixty Brassicaceae sharing the potential to form isothiocyanates. Hence, rapeseed may be only one among many Brassicaceae sharing the potential to form COS during the late stages of growth. COS is also produced from wheat [8] although it lacks the capacity to produce glucosinolates and myrosinase [30].**

**The empirical production function from the comparison of observed and modelled COS non-photosynthetic fluxes (Fig 5C) is suitable for inventorying COS emissions from rapeseed crops at different spatial scales. Rapeseed is mainly grown in Canada, China, the European Union, India and Australia. The total harvested areas represented about 32 million ha in 2015 [31]. Assuming that COS emissions remain invariant all day long, our empirical function, integrated over 3 months, yields a yearly total emission of 0.84 ± 0.13 GgS yr-1 which, as such, does not make a significant contribution to the global budget of COS because the net uptake of COS by soils and vegetation is estimated by ORCHIDEE to be 606 GgS yr-1 [2]. Wheat total harvested areas represent about 215 million ha (https://wheat.org/wheat-in-the-world/). Assuming that rapeseed and wheat share the same capacity of COS production during their late stages of growth, a yearly total emission of 4.7 ± 0.7 GgS yr-1 is obtained. If all C3 crops would share that capacity, a weak assumption, a yearly total emission of 33.3 ± 5.1 GgS yr-1 is obtained.**

L404: “COS emissions” -> “COS concentration enhancements”, to be precise.

**We agree.**

L407: “net non-photosynthetic uptake” -> “net nocturnal uptake”. Daytime soil fluxes are also part of “non-photosynthetic” fluxes, but we do not know their magnitude since they are not measured.

**We agree.**

Fig. 1: Please indicate downwind and upwind sampling locations.

**The wind directions varied from day to day over the three “summers” of survey. Sampling locations were adapted according to the wind direction observed in the field.**

Fig. 3: This plot of concentration time series does not directly inform the main points about fluxes. Suggest offloading it to the Supplement.

**Moved to supplement.**

Fig. 4: Report CO2 fluxes in molar units because COS fluxes are in pmol m^-2 s^-1.

**Done.**

Fig. 5: This is a comparison between observed and simulated nighttime fluxes. They should not be interpreted as the ecosystem budget, unless you have data to show that daytime emissions behave the same as nighttime emissions.

**The legend of Fig. 5, now numbered Fig. 4, has been modified accordingly.**

**Fig 4. comparison between observed and simulated nighttime fluxes at the GIF site…**
