# Supplementary material for: Carbonyl sulfide (COS) emissions in two agroecosystems in central France
Source: PLoS One. 2022 Dec 6;17(12):e0278584. doi: 10.1371/journal.pone.0278584 (PMC9725148; doi:10.1371/journal.pone.0278584)
Supplement: Attachment — Submitted filename: Response to Reviewers.docx [file pone.0278584.s009.docx]

Reviewer #1: The revised manuscript has been greatly improved in clarity and soundness. The description of methods is much easier to follow now. The interpretation of results is put on more solid ground. I only have a few minor comments.

**Answer: The authors thank the referee for the overall positive answer to this study.**

L114–116: “A proxy of the ratio of nocturnal ecosystem fluxes (FCOS/FCO2) was calculated from nighttime vertical gradients of COS and CO2 (GradCOS/GradCO2 from data collected at 5 m and 180 m height)” - It may help to give the readers a reason why you calculate the gradient between 5 m and 180 m instead of that between 5 m and 50 m.

**Answer: An illustration of the vertical distribution of COS at TRN is provided below. Same as S2A Fig, but showing mixing ratios recorded at 50 m (in green) and 100 m (in orange) height.**

**During the 3^rd^ week of June 2020, nocturnal COS losses were also observed at 50 m and, in a lesser extent, at 100 m. Within the nocturnal boundary layer (see z_i_ discussion), 180 m is the sampling height up to which the nocturnal COS drawdown almost never propagates. That is why reference air is taken at 180 m.**

L117: “CO2 flux-gradients” -> “CO2 fluxes and gradients”

**Answer: The text has been updated accordingly.**

L237: “The authors of ref. [23] showed that ...”

**Answer: The text has been updated accordingly.**

L301: “phase” is ambiguous here. Saying that they have opposite signs should suffice.

**Answer: We rephrased this sentence as follows: “At GIF site, the temporal variations in nocturnal ecosystem exchange rates of COS and CO2 simulated by ORCHIDEE have opposite signs (Fig 4A).”**

Fig. 3A: Use English decimal points in the legend.

**Answer: Thank you, this error has been corrected.**

Fig. 4C: The data points don’t appear to follow the prescribed equation very well. Perhaps a non-parametric fit such as local regression would represent the temporal trend of “additional fluxes” more faithfully.

**Answer:** **We would like to highlight that the observed data show a strong dispersion in this figure, limiting the ability of the parametric model to fully fit the data. We agree that a local regression does not require to provide a function to be fitted, however, it requires a large number of data to provide an adequate fitting. To our knowledge, local regressions are more often used with a purpose of smoothing the observed data. Moreover, local regressions usually do not provide a "simple" model, contrary to a parametric function. In this study, we chose to consider a parametric model as we aimed at finding a simple empirical function that could be used to estimate the contribution from the additional source at a larger scale. We think that adding this entire response in the revised version of the manuscript would be a bit “heavy”. We propose the following shorter version of this response: “Here, as we aim at finding an easy-to-use empirical function to describe this difference, May to July data shown in Fig 4C has been fit with a polynomial function of the following form applied from May 1^st^”. The text has been updated accordingly.**

L430–432: "The empirical production function from the comparison of observed and modelled COS non-photosynthetic fluxes (Fig 5C) is suitable for inventorying COS emissions from rapeseed crops at different spatial scales.” - The generality of this “empirical production function” needs to be tested. We don’t know if the temporal features described by this equation apply to other rapeseed fields. The magnitude of the peak emission, the date it occurs, and how quickly it wanes may vary from site to site.

**Answer: Thank you, this is a very pertinent remark. The text has been updated accordingly as follows: “However, because its generality has not been tested yet, it should be used with caution. Indeed, we don’t know if the temporal features described by this equation apply to other rapeseed fields. The magnitude of the peak emission, the date it occurs, and how quickly it wanes may vary from site to site. Nevertheless, rapeseed is mainly grown in Canada, China, the European Union, India and Australia…”**

Reviewer #2: Dear Dr. Belviso, et al.,

OCS fluxes over agricultural fields are an important conundrum in global OCS modeling. Some crops (e.g. rapeseed, investigated here) emit OCS, and some soils emit OCS when hot and dry. A multi-year investigation using multiple methods furthers our understanding of the complexity of crops and OCS. This project is well-conceived and my comments are therefore minor.

**Answer: The authors thank Mary Whelan for the overall positive answer to this study and for the very helpful comments to improve the manuscript.**

Abstract: It might bear mentioning that the GIF site has observations going back to 2014. This would be helpful to alert the reader to the excellent dataset, the length of record in some of the figures, and the multi-year usefulness of this site.

**Answer: The abstract has been updated accordingly as follows: “Second, field observations of COS nocturnal fluxes, obtained by the Radon Tracer Method (RTM) at a sub-urban site near Paris, were used for that same purpose (i.e., a “RTM vs. model” approach of unaccounted biogenic emissions). This site has observations going back to 2014.”**

Data: While I appreciated being able to download the excel spreadsheet of observational data quickly, the excel file itself has no metadata and I’m not sure about the longevity of mycore.core-cloud.net. The data at data.ipsl.fr/respository is obviously preferable, though these files don’t appear to have metadata either. One could say, “the data is described in the paper” which is true. What would be good is to put the citation in the header of the file and maybe (if you are feeling generous) include a brief description of the variables.

**Answer: The data at data.ipsl.fr/repository has metadata, but in XML format which is not very convenient… We will make it available in a new format asap. The excel file has been updated too (https://mycore.core-cloud.net/index.php/s/wUsUpMYrW9FUniz).**

Supplemental: There is no limit to the length of articles for this publication. I’m not sure why we would therefore have a supplemental. Why not have all the figures in one, crisp narrative? The supplemental figures are well-rendered.

**Answer: The first reviewer strongly recommended to have a supplemental and to move more figures to the supplements.**

(Very) minor comments
25: Not yet accounted for.

**Answer: The text has been updated accordingly.**

87-88: Is this a statement that needs to be made in the published work? This almost feels like it should go at the end under acknowledgements, but I don’t know about the shifting requirements of environmental research where you are.

**Answer: Yes, it is mandatory.**

102: Original image of what?

**Answer: This statement is required and will appear in the published article (copyright issues concerning maps).**

107: Is this an archived website that will be there in 20 years? Otherwise, include a brief description.

**Answer: We think so, because ICOS entered the European Strategy Forum on Research Infrastructures (ESFRI) roadmap in 2006, and established the European Research Infrastructure Consortium (ERIC) in 2015. ICOS has established a roadmap for the next 10-20 years.**

115-116: Please write the equation to calculate gradients. Was it really concentration at 5 m – concentration at 180 m? Why collect at 4 heights?

**Answer: Yes, it is really concentration at 5 m minus concentration at 180 m. An illustration of the vertical distribution of COS at TRN is provided below. Same as S2A Fig, but showing mixing ratios recorded at 50 m (in green) and 100 m (in orange) height.**

**During the 3^rd^ week of June 2020, nocturnal COS losses were also observed at 50 m and, in a lesser extent, at 100 m. Within the nocturnal boundary layer (see z_i_ discussion), 180 m is the sampling height up to which the nocturnal COS drawdown almost never propagates. That is why reference air is taken at 180 m. The text has been updated accordingly.**

When people talk about the flux gradient method, normally we think about something a little more complicated, e.g. see section 2.6 of Griffis et al., 2005, <https://doi.org/10.1016/j.agrformet.2005.10.002>.

**Answer: Reviewer 1 also raised this point at the first stage of the review process. In our previous answer, we put the approaches of Rastogi et al. (2018), Kooijmans et al. (2017) and ours in perspective. The major conceptual difference between Rastogi’s approach of biogenic fluxes and ours is that the former investigated the net ecosystem exchange of COS (NEE_COS_ = F_COS_ + S_COS_) from canopy fluxes (F_COS_), measured by the aerodynamic gradient method at the top of the canopy (i.e. between 60 and 70 m), and storage fluxes (S_COS_) which were estimated as the integral of concentration changes over height up to the height of the profile system (i.e., 70 m). Note that in Kooijmans et al. (2017) F_COS_ was measured by the eddy-covariance technique. It is not possible to apply the approach of Rastogi or that of Kooijmans at the TRN site because the 50, 100 and 180 m inlets are located far higher than the typical canopy height and the 5 m inlet much too low. In fact, the TRN tall tower is not designed to specifically investigate biogenic fluxes neither by the aerodynamic gradient method nor by the eddy-covariance technique. The relative vertical gradients of COS measured at TRN between 5 and 180 m can be used to assess local exchange rates provided that the highest inlet height remains within the nocturnal boundary layer (NBL) during the entire night as demonstrated previously (see discussion about z_i_).**

117-121: [21; 4[ (UTC)? This sentence needs to be revised. There are too many ideas in it. This makes it sound like TRN was only used for nocturnal gradients.

**Answer: That is correct, TRN data was essentially used for spring and summer nocturnal gradients.**

190, 192: You could put in the latin names for plant scientists of the future.

**Answer: The text has been updated accordingly as follows: Winter rapeseed (*Brassica napus*, Blackbuzz variety) was sown on September 5th, 2020 and harvested August 6th, 2021. A mixture of four varieties of winter wheat (*Triticum aestivum*; Renan, Gwenn, LG Absalon, and Chevignon varieties) was sown on October 31st, 2020 and harvested August 14th, 2021.**

197-198, 254: Is this a statement required of the funding source?

**Answer: Our original plans were to document for three years (2019 to 2021) horizontal gradients at GIF and vertical gradients at TRN with the same success. Unfortunately, in 2020 we faced the COVID-19 lockdown and the failure of the COS laser (annus horribilis…). That is why data between both sites are not that much homogeneous.**

200: It might be cultivars, not cultures.

**Answer: The text has been updated accordingly.**

232: Quotes are unnecessary /confusing here.

**Answer: Quotes have been removed.**

247: How do you get a single land use type from a pair of concentrations?

**Answer: Because the highest inlet height remains within the NBL during the entire night in late spring and summer (cf. z_i_ discussion and S1 Fig), the nocturnal vertical gradients are driven by local processes so by a single land use type. This sentence has been revised as follows: Thus (1) the 180 m inlet did not sample air from the residual layer (RL) by night, in support of Pal et al. (2015)’s scenario B ([23], cf. Fig. A1), and (2) the concentration gradients between the two levels indicate a single land use type, i.e. that they are driven by local processes.**

278-281: There are logical leaps to get to this statement. They just need to be spelled out.

**Answer: To get to this statement, we added two sentences (in red in the following): “Nocturnal ecosystem fluxes of COS and CO_2_ simulated by the ORCHIDEE model are strongly correlated throughout the growing season, except during a heatwave which lasted a few days in July 2019 and resulted in enhanced soil production of COS (Fig 3B).** **This strong correlation describes a proportionality between the CO_2_ and COS fluxes that remains constant over all summer months, as opposite to what is found for the concentration gradients (Fig 3A) and illustrated by a different linear regression each month. If a decrease in ecosystem efficiency to absorb COS was simulated until July, followed by a recovery in August, we would not expect a single linear regression to be able to fit the simulated COS versus CO_2_ fluxes. Hence, this simulated COS exchange, i.e., summed changes in nocturnal stomatal conductance and soil fluxes, failed to reproduce the decrease in the net sink of COS at TRN during the spring-summer 2019 and its recovery at the end of summer.”**

298: It might be good to explain what you think the emissions episodes are caused by here.

**Answer: As a transition to the next section, we have added the following sentence: “****Model simulations have been used to assess their origin”.**

311: The emission is unidentified, but this is the results and discussion section. There are plenty of candidates in the literature you could mention. Why do this? It will inspire other folks to try and figure it out and they can start with a hypothesis to test.

**Answer: This is the legend of Fig 4. We identified an additional source of COS yet unidentified at this stage of the presentation of the results. The possible candidates that have been identified in the literature are discussed in the following parts, with the related references.**

352-354: This conclusion needs to be given greater prominence.

**Answer: The sentence has been revised as follows: “These observations are of crucial importance because they very likely tell that the COS source is in the plant not in the soil.”**

366-367: Is the difference in uptake related to a difference in GPP?

**Answer: Unfortunately, GPP was not measured at the GIF site.**

379-382: I thought you just demonstrated that there’s a missing plant-based process not accounted for by models?

**Answer: We refer here only to the study of Maseyk et al. for which we are unaware of any comparison between simulated and observed fluxes.**

385: Kadmiel Maseyk can send you the data so you do not have to read it off of Fig 1.

**Answer: Unfortunately, we got no answer to our request for data about a year ago.**

374-419: This paragraph is a beast. It would be easier to understand what is going on by breaking it up into more digestible pieces.

**Answer: Sorry for that. It is now split into three paragraphs.**

400: Crops seem by their nature to be perturbed?

**Answer: The sentence now reads: “That is, our surveys of agricultural crops were carried out in conditions not perturbed by fungal infections.”**

418-419: The OCS emissions at Bondville can account for 25% or so of the total flux.

**Answer: The end of the last paragraph now reads: “Maize would deserve special attention because it has been suggested that emission of COS from a largely senesced maize field in Bondville (USA) can disguise any leaf uptake of this gas at the end of the season [28]. The COS emissions at Bondville can account for 25% or so of the total flux (Mary Whelan, pers. com. dated October 2022).”**

410: I would do a find-and-replace for OCS to make sure it’s all COS (or all OCS).

**Answer: It is all COS except in the reference list.**

428-429: It seems like this paragraph should start, “The COS production path in plants is not completely known.” The way it starts now, it seems as though the production path has extensive research, but then we end by saying we have no idea what’s going on with wheat.

**Answer: Thank you, the paragraph has been updated accordingly.**

446-459: This conclusion is okay, but is anemic compared to the colossal effort and an excellent dataset. Where do you want it to go now?

**Answer: The second half of the conclusion as been rewritten as follows: “A COS empirical production function is proposed which generality needs to be tested in other rapeseed fields especially in terms of duration and magnitude of the peak emission. The role of other plants of the “cabbage order” needs also to be addressed experimentally in the fields with dynamic chambers or by eddy covariance. Although our field observations support qualitatively those of [8], some inconsistencies remain as to the relative importance of COS emissions from senescent wheat at the plot scale on both sides of the Atlantic. For now, it is suggested that emissions from rapeseed alone and C3 crops in general cannot account for the missing source of COS in the global budget of this gas”.**

Thanks for moving this idea forward,
Mary Whelan
